# Reports of Cases Occurring in the Medical Ward of the Buffalo Hospital

**Published:** 1857-10

**Authors:** A. W. Nichols

**Affiliations:** Assistant to Chair of Clinical Medicine, University of Buffalo


ART. IV.—*Reports of Cases treated in the Male Medical Ward of the Buffalo Hospital of the Sisters of Charity*, AUSTIN FLINT, M. D., *Attending Physician, during the College Session of* 1856–7, *being from October* 1, 1856 *to February* 18, 1857. Reported by A. W. NICHOLS, M. D., Assistant to Chair of Clinical Medicine, University of Buffalo.

### No. VII. *Acute Lobar Pneumonitis.*

During the past winter and spring, several cases of pneumonitis occurred at the hospital, presenting, during the course of the disease, many interesting and important points pertaining to the signs and symptoms, duration and treatment, of this disease. Seven of these cases were instances of simple acute lobar pneumonitis; and two others were instances of inflammation of the lung supervening upon probable tuberculous deposit.

At the risk of tiring the reader, we will at this time enter into a brief discussion of certain signs and symptoms presented ordinarily in the course of pneumonitis, and present a few diagnostic features of this disease; leaving for a subsequent article, the more interesting subject of the treatment of inflammation of the lungs.

In simple acute pneumonitis occurring during adult life, it is an interesting and important fact, that the inflammation is almost always confined, at first, to the lower lobe of either lung. The signs consequently presented on physical examination of the chest, are very different and distinctive according as we conduct the exploration over the affected or unaffected portions of the lung on the same side of the chest. Is it practicable, then, in the great majority of cases, to demonstrate by means of a physical examination of the lungs, the precise limits of the affected tissue? It is, and with comparative ease. The signs by which we are enabled to draw a line of demarkation on the chest, and to assert that *here* is inflamed lung and *there* healthy lung, are readily recognized and are presented in almost every case of this variety of pneumonitis during each stage of its course. These physical signs are furnished by recourse to percussion, to auscultation of the respiration, of the voice, and of the whisper, and to inspection.

On percussing over the affected portion of the chest, namely, the lower lobe of one lung, it is highly important to remember that, in place of flatness or dullness, whhich signs might be expected from a lung more or less solidified, there is frequently present a tympanitic sound more or less intense. This resonance may be more intense and clearer than that produced by percussing over a corresponding part on the unaffected side of the chest, yet it is *high* in pitch and *metallic* in quality. The tympanitic resonance is more commonly found anteriorly and laterally, than over the scapula below the ridge: in the latter situation, we usually meet with dullness or flatness. Occurring in cases of pneumonitis affecting the lower lobe of the left lung, the tympanitic resonance may be due to distension of the stomach with gas; but, tympanitic resonance, not so acute in pitch nor of such metallic quality, exists in the above location, when there is no tympanitic sound on percussion over the stomach. Over the upper lobe on the affected side, it is necessary to observe, that the percussion-resonance is frequently modified; and that there is often so much modification of the vesicular resonance, as to give rise to that resonance described as "*vesiculo-tympanitic.*"* Now, it is only necessary for our present purpose, to know that at a certain line running diagonally across the chest on the affected side, the percussion-resonance from below upward suddenly changes at this line from tympanitic, and is found to become more or less vesiculo-tympanitic. This change in the quality of the resonance, is so abrupt usually, that we are able to trace with great accuracy the lines of demarkation between healthy and morbid lung.

But, to show still farther the accuracy of physical signs, and to exhibit the correlative value of some of them, attention may be directed to the signs derived from auscultation, in regard to the above point. In the first place, we may state, that "it not infrequently happens that, over the small portion of the lower lobe which extends in front, auscultation fails to detect any morbid phenomena." "Posteriorly and laterally, if the stethoscope be employed by passing the instrument over successive portions of the chest from above downward, the change from the vesicular murmur to the bronchial respiration is found to be abrupt, not gradual. If the line indicating the situation of the interlobar fissure have been already traced by the change in the percussion-sound, the transition from the vesicular murmur to the bronchial respiration will be found to take place on the same line. The limits of solidification may thus be defined by auscultation as well as by percussion, and it is in some cases easier to trace the boundaries by means of the former

* Flint on Respiratory Organs, p. 106. Vesicular respiration not abolished, but modified by tympanitic qualities. N.

than by the latter method. On the back, the characters of the bronchial respiration are shown in striking contrast by auscultating alternately above and below the spinous ridge of the scapula."*

Furthermore, the sounds elicited by the articulated and whispered words, furnish additional and confirmatory evidence in regard to the accuracy of the line drawn by means of the foregoing signs of percussion and auscultation, and denoting the inter-lobar fissure. Below this line, bronchophony usually exists; above, exaggerated vocal resonance. But, the *puff* or *souffle* of the whispered words, often becomes as distinctive as any of the previous physical signs; in other words, the change in the character of the sound above and below the line of inter-lobar fissure, appear more abrupt and is more marked. This is noticed not only in front, but also behind over the scapula. Applying the stethoscope over successive portions of the affected side of the chest, from below upward, the sound of the whispered words is found to be quite intense and *high* in pitch; but, at a certain line, in front and behind, the souffle becomes changed abruptly, and is now low and vesicular. In several cases, this alteration in the character of the sound has been so sudden, that the souffle obtained from that portion of the chest included in the end of the stethoscope was entirely different from that obtained when the instrument was moved in a contrary direction, above or below the line, the smallest appreciable distance. This sign may then frequently become of importance on account of its delicacy. In every one of these cases in which regard was paid to this sign, it confirmed the correctness of the line previously drawn by the foregoing physical signs.

The line of inter-lobar fissure commencing in front, runs upward and backward, from a short distance within and below the nipple, to about the vertebral extremity of the spine of the scapula.

It will be unnecessary to detail the signs furnished in those cases in which the line of inter-lobar fissure was demonstrated. We will observe, however, that the foregoing physical signs referring to changes in the percussion-resonance, and in the sounds of the respiration, the voice, and the whisper, were so distinctive in four of the nine cases, that this line was drawn on the chest, with ease and accuracy. In one of these cases, the disease had nearly run its course, and the affected lung was returning to a state of health; the examination having been made *three weeks* after date of attack.

In the remaining five cases, this line could not be drawn for various reasons: in one case, the entire lung on one side, was affected; in two cases, no

* Flint, op. cit., part ii, chap. iii.

observations were made in reference to this point; and in two cases, the disease, in all probability, occurred in connection with tuberculosis, and was not confined to a single lobe.

We have a few remarks to offer, respecting the *crepitant and sub-crepitant râles*, and the crepitant râle redux. Some observers have denied that this râle exists in the majority of cases of pneumonitis, and consequently have thrown doubt on the value of the crepitant râle as a pathognomonic sign.

In these nine cases, the crepitant râle was heard in *five* cases; the sub-crepitant ràle in *three;* the crepitant râle redux in *one;* and no râles in one case. The crepitant râle was noticed in these five cases as follows: in one, seven days after the attack, at end of a forced inspiration, in the lower axillary region, and not elsewhere; in one, nine days after date of attack, just below the angle of the scapula; in one, five days after date of attack, over scapula below ridge; in one, eight days after attack, faint and at end of inspiration, below scapula. In another case, the sub-crepitant râle had previously existed, accompanied with considerable expectoration of blood and mucus; as the expectoration diminished and became less bloody, the crepitant râle took the place of the sub-crepitant, being heard well-marked and at the base of the lung behind; it was first observed eight days after date of attack; and was not heard after ten days from date of attack.

In three of the four cases in which the crepitant râle was not heard, the disease had been existing several days prior to the admission of the patient: and when the chest was examined, the physical signs showed the lung to be returning to a condition of health. In the remaining case, the disease had existed eight days, (in connection with tuberculosis, probably,) and no râles were heard. So that, the fact of the occurrence of the crepitant râle in only five of nine cases, does not go to disprove the great value of this sign in diagnosis, as regards its frequency. But, these cases show—if any thing—that this râle is generally heard in the first stage of the inflammation, and before there has been a very great amount of solidification; and that this râle may vary greatly in intensity and in continuance: being sometimes very loud, near the ear, and occupying a great portion of the inspiration; and at other times, faint, remote, and heard only at the end of a forced inspiration. Besides, it requires, not infrequently, a careful examination of the chest to discover its situation; generally existing over or just below the scapula, and sometimes only during forced inspirations.

In regard to the frequency of the crepitant ràle redux, this sign was heard in a single case. The disease had existed three weeks, and the physical signs furnished evidence of the lung returning to a state of health: a well-marked crepitant râle was heard at the end of a forced inspiration just below the scapula.

In reference to the continuance of the crepitant râle through the entire duration of the disease, or its disappearance and subsequent return as the crepitant râle redux, Dr. Flint states, that "the observer who seeks by daily explorations during the career of pneumonitis to verify the *crepitant râle redux*, will very often meet with disappointment. The crepitant râle, as just stated, may continue through the whole course of the disease. It may disappear and reäppear at irregular intervals. I have known it to become more marked after the lapse of several days, than at an early period in the disease."

The sub-crepitant râle was found to exist in three of the nine cases: in one, there was a large quantity of bloody expectoration, on the decrease of which, on the fifth day of the attack, the sub-crepitant râle gave place to a true crepitant râle; in the other two cases, the disease had existed seven and twelve days, respectively; and in both cases, there appeared to be a considerable amount of expectoration. "The sub-crepitant râle is much more likely to occur at a late period in the disease, during the progress of resolution. Developed under these circumstances it is, in fact, the returning crepitant râle of Laennec. But, its appearance is by no means constant."

Inspection sometimes furnishes signs of more or less importance, in pneumonitis. We refer more particularly to the expansive movements of the two sides of the chest, excepting those cases of pneumonitis in which the movement of the affected side is instinctively restrained on account of the severity of the pain, these cases furnished evidence in favor of the opinion that the affected side, in simple acute lobar pneumonitis, does not expand equally with the healthy side. Grissolle is of the opinion that there is no disparity between the two sides in regard to the amount of expansion. Dr. Flint states, that "a disparity in expansion-movement at the inferior portion of the chest is sometimes obvious, and in other instances not apparent." "It is more marked if the breathing be labored, or voluntarily forced." Again, "the deficient expansion of the affected side when pain has ceased to be a prominent symptom, in other words in the second stage, is attributable to the augmented size of the lung, and the loss of its contractility."

Of these seven cases of simple acute lobar pneumonitis, there was a greater amount of expansion of the healthy side, in five cases; this was first observed as early as the second day in one case; and in another instance not until the ninth day. It was noticed in all the cases, on the first examination of the chest. In the two cases probably complicated with tuberculosis, the difference in mobility of the two sides, was observed at an early period of the inflammation. In all of these cases, the disparity was rendered more evident on forced breathing; and in most of them could be detected readily during ordinary breathing. In a few instances, not only were the superior and inferior costal types of breathing greater on the unaffected side, but likewise the abdominal type.

An interesting question here arises: how long after the cessation of the inflammation, does the affected lung furnish any physical signs retrospective of pneumonitis? We are able to furnish the results of examinations made in two cases, a considerable time after the date of the attack. In one case, the disease had pursued its course and terminated in recovery without any medication, and under very unfavorable hygienic conditions; an exploration of the chest made twelve days after date of attack, revealed signs distinctive of lung returning from a state of solidification to health. In the other case, the examination was made twenty-one days after date of the attack. The physical signs, in the latter case, were as follows: no apparent disparity in mobility on the two sides; the percussion-resonance over the lower lobe of left lung, (the affected lobe,) was still tympanitic, and the line of inter-lobar fissure could be traced readily by the abrupt change in the character of the resonance, from below upward; a true crepitant rále redux was discovered at the end of a forced inspiration, over the left side of the chest below the scapula; broncho-vesicular respiration existed over the left inter-scapular space, but ceased abruptly at the level of the third dorsal vertebræ and opposite the vertebral extremity of the spine of the scapula; the wispering souffle was still distinct and high-pitched on the left side below scapula and not appreciable on the right side. As far as the above case is concerned, it may be practicable to determine the prior existence of pneumonitis, after a lapse of three weeks from the time of attack.

We have presented a few points of interest relating to the physical signs in pneumonitis, as derived from a revision of nine cases. Most of the signs which have been the subjects of this discussion, have been matters of dispute with different observers, and are still so to some extent. These cases have been introduced, merely, to show the facility with which we are able to determine the precise extent of the affected lung in pneumonitis. They may serve, likewise, as additional testimony in favor of the presence of certain physical signs in the great majority of cases of simple acute lobar pneumonitis.

